# Outcomes for patients with high-risk ER-positive, HER2-negative early-stage breast cancer: a Danish real-world study

**DOI:** 10.2340/1651-226X.2025.44003

**Published:** 2025-11-12

**Authors:** Ida Christine Jacobsen, Tobias Berg, Maj-Britt Jensen, Ann Søegaard Knop

**Affiliations:** a Department of Oncology, University Hospital Copenhagen, Rigshospitalet, Denmark; b Danish Breast Cancer Group, Department of Oncology, University Hospital Copenhagen, Rigshospitalet, Denmark

**Keywords:** Early breast cancer, receptors, estrogen, female, real-world, outcome, adjuvant cyclin-dependent kinases

## Abstract

**Background:**

While adjuvant CDK4/6 inhibitors (abemaciclib and ribociclib) have improved invasive disease-free survival (iDFS) in ER-positive, HER2-negative early breast cancer (EBC) in the MonarchE and NATALEE trials, their real-world applicability in Denmark remains unclear. This study evaluates Danish patients meeting comparable high-risk criteria and their outcomes, hypothesizing that a substantial proportion could benefit from additional adjuvant treatment options.

**Methods:**

Patients with ER-positive, HER2-negative EBC, diagnosed 2014–2019, who met MonarchE and/or NATALEE eligibility, were included and categorized as intermediate or high risk corresponding to trial definitions. Outcomes were overall survival (OS), iDFS, cumulative incidence of distant recurrence-free survival (DRFS) events and endocrine therapy adherence.

**Results:**

Of all new cases of EBC, approximately 31% were included. Of 5,788 patients, 59.1% were intermediate risk and 40.9% high risk. Five-year OS and iDFS were lower in high-risk than intermediate-risk patients (84.5% vs. 91.9% and 76.2% vs. 85.7%, respectively), and cumulative DRFS event rates were higher (18.5% vs. 8.9%). High-risk patients more often received chemotherapy, yet nonchemotherapy subgroups in both risk categories had worse outcomes. Endocrine therapy adherence at 5 years was 77%.

**Interpretation:**

A considerable proportion of Danish EBC patients meet high-risk criteria similar to CDK4/6 inhibitor trial populations and experience inferior outcomes despite standard therapy of antihormone treatment +/- chemotherapy. Our real-world data underscore the need for more effective and less toxic adjuvant therapies such as CDK4/6 inhibitors.

## Graphical abstract

**Figure:**
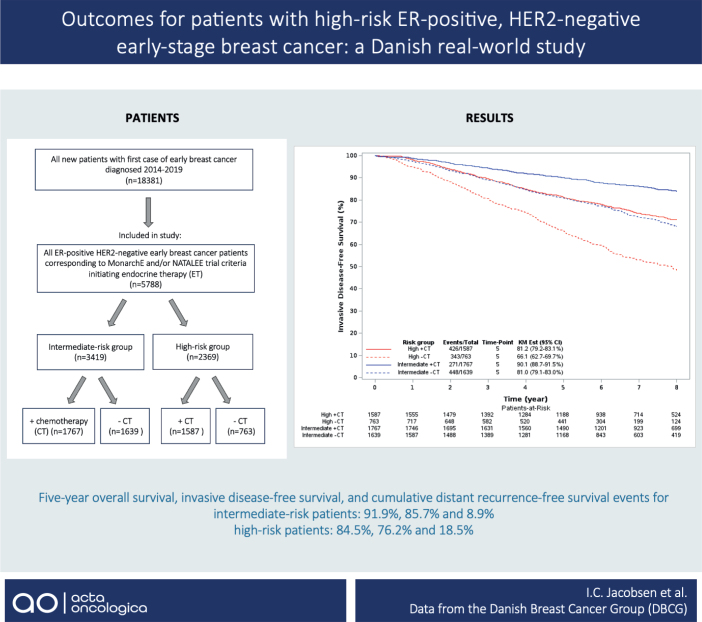

## Introduction

Adjuvant treatment strategies for patients with hormone receptor-positive breast cancer are advancing. The FDA and EMA have approved abemaciclib and ribociclib, cyclin-dependent kinase 4 and 6 (CDK4/6) inhibitors, in combination with endocrine therapy (ET) as adjuvant treatment to patients with hormone receptor-positive, human epidermal growth factor receptor 2 (HER2)-negative early breast cancer (EBC) to reduce the risk of recurrence in addition to its use in the treatment of metastatic disease. In the MonarchE trial (NCT03155997), the addition of 2 years of abemaciclib to ET improved the 5-year invasive disease-free survival (iDFS) by 7.6%, and further gains in iDFS have been seen with additional follow-up [1, 2]. In the NATALEE trial (NCT03701334), the addition of ribociclib for 3 years showed a relative reduced risk of recurrence by 25% [3] and an improved iDFS. At the latest 4-year landmark analysis, the absolute improvement in iDFS rates was increased from 2.7% at 3 years to 4.9% at 4 years, showing benefit even after completion of ribociclib [4]. A consistent benefit was observed across subgroups, including stage II and III disease and node-positive and node-negative disease. Overall survival (OS) follow-up is ongoing but in favor of the combined treatment.

The European Medicines Agency (EMA) approved abemaciclib in April 2022 for adjuvant treatment of high-risk, early-stage breast cancer and ribociclib in November 2024 for stage IIA–III disease meeting specific high-risk criteria. In February 2025, the Danish Medicines Council recommended CDK4/6 inhibitors for high-risk patients and, from September 2025, partially extended this to intermediate-risk patients (N0 and T3 or T2/grade III). The Council further clarified that patients with macrometastases in two sentinel nodes (SNs), in the absence of axillary dissection, may also receive adjuvant CDK4/6 inhibitors.

All premenopausal patients with high-risk criteria are recommended ET and chemotherapy, provided they can tolerate it. Since 2013, Denmark has used a prognostic standard mortality rate index (PSI) to guide adjuvant systemic therapy in postmenopausal women with ER-positive, HER2-negative EBC, with N0 and N1 disease. A higher score indicates a higher excess mortality risk, and chemotherapy is recommended in addition to ET if the PSI is high or intermediate and with luminal B subtype accessed by the microarray 50 gene expression classifier (PAM50) [5].

The aim of this study is to present the proportion of patients with EBC qualifying for adjuvant CDK4/6 inhibitors and their outcomes in terms of OS, iDFS, cumulative incidence of distant recurrence-free survival (DRFS) events, and adherence to ET based on data from the Danish Breast Cancer Group (DBCG) to discuss the potential benefit of adding CDK4/6 inhibitors in the treatment of patients with intermediate- and high-risk disease.

## Methods

### Data in DBCG

Data on diagnosis, pathology, demographics, treatment, and follow-up are registered prospectively in the clinical database of DBCG. Chemotherapy (CT) is recorded with specific dose and date for each agent administered, and ET is recorded with the type of agent every 6 months by the attending physician. Patients are followed to a first event or for a total of 10 years. Date and location of recurrence, contralateral breast cancer (CBC), second non-breast malignancies (non-BC), and death are registered. The Danish Civil Registration System is linked to the DBCG by personal identification number to secure complete follow-up on vital status and emigration.

### Study design

The current study was an observational, retrospective, nationwide population-based study covering patients from all departments of oncology in Denmark. All patients registered in the DBCG database fulfilling the inclusion criteria were included.

The study was registered in the Capital Region’s research overview (P-2024-18094) and approved by the Centre for Health of the Capital Region (R-24080812).

### Population

Patients diagnosed between 1^st^ of January 2014 and 31^st^ of December 2019 with an ER-positive, HER2-negative EBC were included if they received ET and fulfilled at least one of the following criteria: Tumor size T3-T4, nodal status N2-N3, T2 if N1 or malignancy grade III, T0-T1 if N1mac (macrometasis), T1 if N1mic (micrometastasis) and malignancy grade III. The cohort was divided into two subgroups: Intermediate risk and high risk. The high-risk subgroup comprised T4, N2-N3, T3N1, or N1GradeIII. The intermediate-risk subgroup comprised the rest. Supplementary Table 1 shows the subgroups in the current study in relation to NATALEE and MonarchE.

ER- and HER2-status by surgical specimen was determined at up-front surgery or biopsy. For patients with up-front surgery, tumor size was determined by surgical specimen and lymph node status by SN procedure and/or axillary dissection. For patients with preoperative systemic therapy, tumor size was determined by ultrasound measurement and nodal status primarily on fine-needle aspiration (FNA) axilla, with a minor part having up-front SN information.

### Outcomes

OS was defined as the time from the date of diagnosis to the date of death of any cause. IDFS was defined as the time from the date of diagnosis to the date of first event of ipsilateral invasive breast cancer recurrence or regional invasive breast cancer recurrence (LR), distant recurrence (DM), CBC, non-BC, or death attributable to any cause. Cumulative incidence of DRFS events was defined as the time from date of diagnosis to date of first event, which included DM and Death, with LR, CBC, and non-BC as competing events.

Discontinuation of ET was calculated from the date of first ET until registration of the end of treatment within 4.5 years (discontinued), censoring patients off the study within 6 months of the last recorded ET, with the end of ET after 4.5 years of treatment, or at the last date of ET with missing information on the complete treatment course. Discontinuation was determined based on the clinical registrations to the DBCG.

### Statistical analysis

The Kaplan-Meier method was used to estimate OS, iDFS, and adherence to ET. Follow-up was estimated by the reverse Kaplan-Meier method. DRFS was estimated by cumulative incidence in the presence of competing risk. Data analyses were performed using SAS EG 8.4.2.22.

## Results

**Figure 1:**
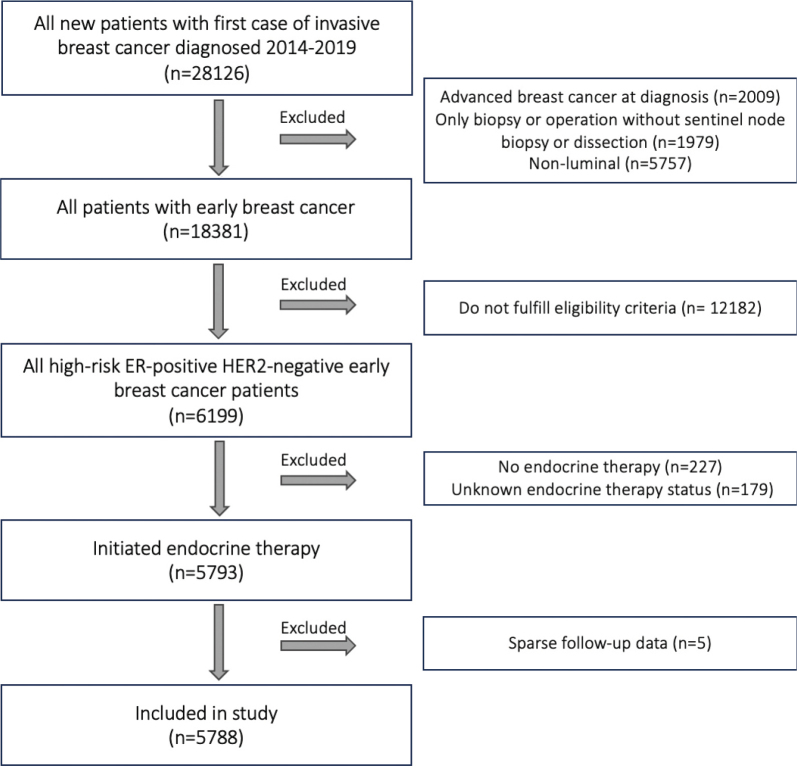
Flowchart of the study.

From 2014 to 2019, a total of 28,126 women were diagnosed with breast cancer in Denmark, of which 18,381 were EBC. Of these, 6,199 patients had ER-positive, HER2-negative EBC with high-risk characteristics, of whom 411 were excluded due to no or unknown ET status or little follow-up data (Figure 1). Our study population thus included 5,788 patients, with 3,419 (59.1%) in the intermediate-risk group and 2,369 (40.9%) in the high-risk group. The median age was 62 years, most had ductal carcinoma (79.9%), and 72.8% were postmenopausal (Table 1). Expected observed differences in the two risk groups relate to tumor size and nodal stage, as well as a higher malignancy grade in the high-risk group.

**Table 1:** Patient characteristics of the intermediate- and high-risk groups.

Total	Intermediate 3,419 No. (%)	High 2,369 No. (%)	All 5,788 No. (%)
**Age**			
Median [IQR]	62 [51;72]	63 [51;74]	62 [51;73]
< 50 years	652 (19.1)	494 (20.9)	1,146 (19.8)
50–59 years	876 (25.6)	502 (21.2)	1,378 (23.8)
60–69 years	836 (24.5)	529 (22.3)	1,365 (23.6)
70–79 years	777 (22.7)	588 (24.8)	1,365 (23.6)
≥ 80 years	278 (8.1)	256 (10.8)	534 (9.2)
**Menopausal status**			
Pre	929 (27.2)	644 (27.2)	1,573 (27.2)
Post	2,490 (72.8)	1,725 (72.8)	4,215 (72.8)
**Nodal status**			
N0	671 (19.6)	116 (4.9)	787 (13.6)
Nmic	280 (8.2)	216 (9.1)	496 (8.6)
N1	2,468 (72.2)	925 (39.0)	3,393 (58.6)
N2	0 (0.0)	737 (31.1)	737 (12.7)
N3	0 (0.0)	375 (15.8)	375 (6.5)
**Tumor size**			
T1	1,413 (41.3)	638 (26.9)	2,051 (35.4)
T2	1,779 (52.0)	930 (39.3)	2,709 (46.8)
T3	227 (6.6)	452 (19.1)	679 (11.7)
T4	0 (0.0)	349 (14.7)	349 (6.0)
**Histologic type**			
Ductal	2,769 (81.0)	1,853 (78.2)	4,622 (79.9)
Lobular	525 (15.4)	467 (19.7)	992 (17.1)
Other	117 (3.4)	48 (2.0)	165 (2.9)
Malignant, NOS	8 (0.2)	1 (0.0)	9 (0.2)
**Malignancy grade**			
I	820 (24.9)	287 (12.4)	1,107 (19.7)
II	1,982 (60.2)	1,056 (45.5)	3,038 (54.1)
III	466 (14.1)	963 (41.5)	1,429 (25.5)
Unknown	26 (0.8)	14 (0.6)	40 (0.7)
Total*	3,294	2,320	5,614
**Chemotherapy**			
Yes	1,767 (51.7)	1,587 (67.0)	3,354 (57.9)
NACT	436 (24.7)	227 (14.3)	663 (19.8)
Adjuvant	1,331 (75.3)	1,360 (85.7)	2,691 (80.2)
No	1,639 (47.9)	763 (32.2)	2,402 (41.5)
Unknown	13 (0.4)	19 (0.8)	32 (0.6)
Type of ET			
**Tamoxifen**	561 (16.4)	350 (14.8)	911 (15.7)
Aromatase inhibitor	2,317 (67.8)	1,649 (69.6)	3,966 (68.5)
Sequential	541 (15.8)	370 (15.6)	911 (15.7)

* Frequency missing = 174 (malignancy grade only for ductal and lobular histologic type).

IQR: interquartile range; mic: micrometastatic; NOS: not otherwise specified; NACT: neoadjuvant chemotherapy; ET: endocrine therapy.

In the intermediate-risk group, 51.7% received CT, compared to 67.0% in the high-risk group. Table 2 shows patient characteristics by chemotherapy status, including differences in tumor size, nodal status, and malignancy grade in the four subgroups and overall revealing a lower median age in those receiving CT in addition to ET (52 years in the intermediate-risk group and 56 years in the high-risk group) compared to those not receiving CT (72 and 77 years, respectively). Most patients in the intermediate-risk group who did not receive chemotherapy were postmenopausal (97.1%) (Table 2) and were allocated to treatment based on their PSI-score. The majority had been allocated to ET alone (83.4%), whereas only 40.9% of high-risk patients who did not receive CT were originally allocated to ET alone. For those allocated to ET alone, the median age was 73 years (IQR 67–78) and 77 years (IQR 72–81) in the intermediate- and high-risk groups, respectively (data not shown).

**Table 2:** Patient characteristics of the intermediate- and high-risk groups +/- chemotherapy (CT).

Total*	Intermediate – CT 1,639 No. (%)	Intermediate + CT 1,767 No. (%)	High – CT 763 No. (%)	High + CT 1,587 No. (%)
**Treatment allocation**				
ET	1,367 (83.4)	10 (0.6)	312 (40.9)	1 (0.1)
ET+CT	235 (14.3)	1,740 (98.5)	427 (56.0)	1,548 (97.5)
Outside protocol	37 (2.3)	17 (1.0)	24 (3.1)	38 (2.4)
**Age**				
Median [IQR]	72 [65;78]	52 [47;59]	77 [70;81]	56 [48;66]
< 50 years	27 (1.6)	624 (35.3)	18 (2.4)	470 (29.6)
50–59 years	147 (9.0)	725 (41.0)	42 (5.5)	455 (28.7)
60–69 years	494 (30.1)	337 (19.1)	109 (14.3)	414 (26.1)
70–79 years	694 (42.3)	81 (4.6)	344 (45.1)	243 (15.3)
≥ 80 years	277 (16.9)	0 (0.0)	250 (32.8)	5 (0.3)
**Menopausal status**				
Pre	47 (2.9)	880 (49.8)	30 (3.9)	604 (38.1)
Post	1,592 (97.1)	887 (50.2)	733 (96.1)	983 (61.9)
**Nodal status**				
N0	237 (14.5)	434 (24.6)	96 (12.6)	20 (1.3)
Nmic	155 (9.5)	125 (7.1)	87 (11.4)	129 (8.1)
N1	1,247 (76.1)	1,208 (68.4)	274 (35.9)	639 (40.3)
N2	0 (0.0)	0 (0.0)	203 (26.6)	530 (33.4)
N3	0 (0.0)	0 (0.0)	103 (13.5)	269 (17.0)
**Tumor size**				
T1	792 (48.3)	613 (34.7)	143 (18.7)	490 (30.9)
T2	792 (48.3)	982 (55.6)	273 (35.8)	650 (41.0)
T3	55 (3.4)	172 (9.7)	108 (14.2)	337 (21.2)
T4	0 (0.0)	0 (0.0)	239 (31.3)	110 (6.9)
**Histologic type**				
Ductal	1,341 (81.8)	1,416 (80.1)	581 (76.1)	1,261 (79.5)
Lobular	228 (13.9)	296 (16.8)	161 (21.1)	300 (18.9)
Other	70 (4.3)	47 (2.7)	21 (2.8)	25 (1.6)
Malignant, NOS	0 (0.0)	8 (0.5)	0 (0.0)	1 (0.1)
**Malignancy grade**				
I	496 (31.6)	319 (18.6)	108 (14.6)	179 (11.5)
II	882 (56.2)	1,094 (63.9)	350 (47.2)	696 (44.6)
III	190 (12.1)	275 (16.1)	281 (37.9)	676 (43.3)
Unknown	1 (0.1)	24 (1.4)	3 (0.4)	10 (0.6)
Total**	1,569	1,712	742	1,561

* Frequency missing = 32 (CT status unknown).

** Frequency missing = 172 in addition to the 32 already missing (malignancy grade only for ductal and lobular histologic type).

ET: endocrine therapy; IQR: interquartile range; mic: micrometastatic; NOS: not otherwise specified; NACT: neoadjuvant chemotherapy. Malignancy grade unknown is for nonductal and nonlobular cancers.

The estimated median potential follow-up for OS was 8 years and 2 months. At 5 years, the OS rate was 91.9% (95% confidence interval [CI]: 91.0–92.8) in the intermediate-risk group and 84.5% (95% CI: 83.1–86.0) in the high-risk group (Figure 1a).

**Figure 2:**
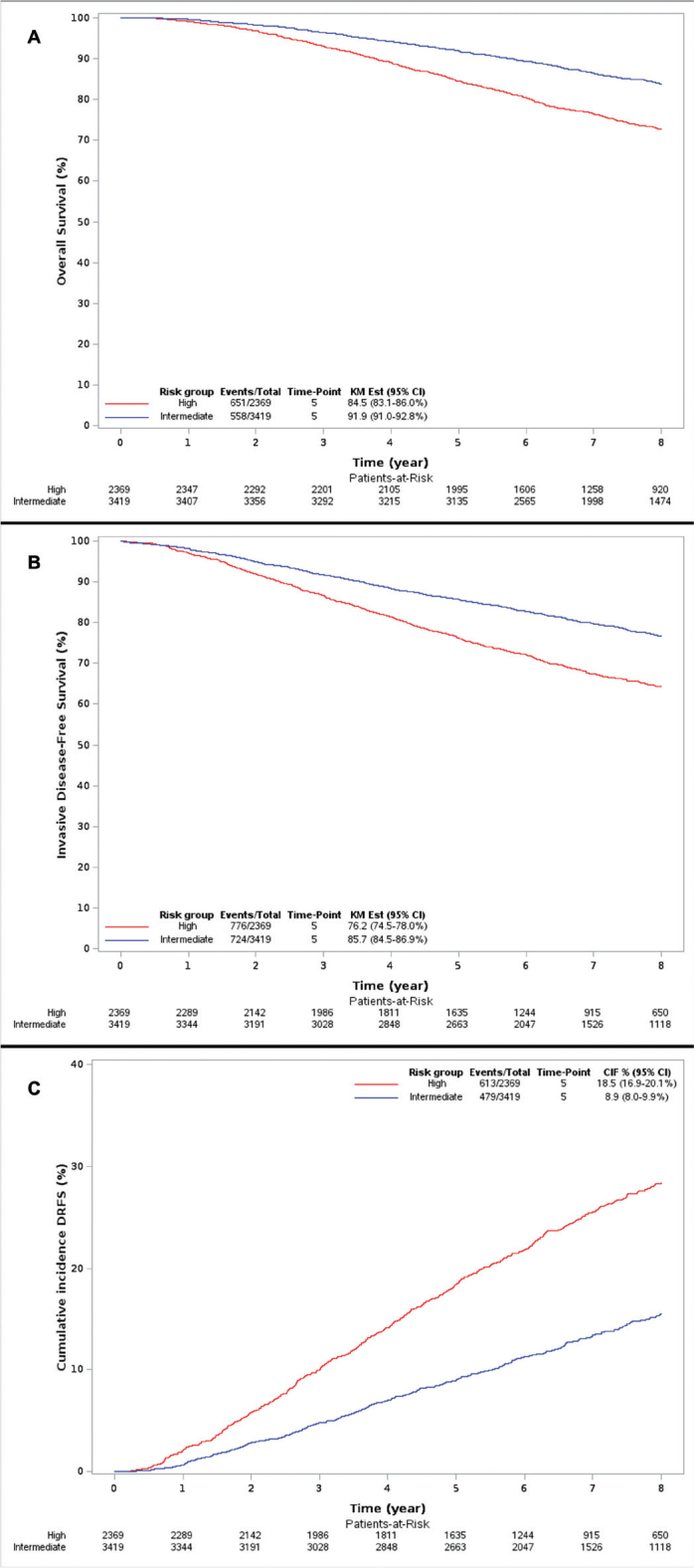
Overall survival (A), invasive disease-free survival (B), and cumulative incidence of distant recurrence-free survival events (C) in the intermediate- and high-risk groups.

The estimated median potential clinical follow-up was 7 years and 6 months. For the intermediate- and high-risk groups, the iDFS rates were 85.7% (95% CI: 84.5–86.9%) and 76.2% (95% CI: 74.5–78.0%), and cumulative DRFS event rates were 8.9% (95% CI: 8.0–9.9) and 18.5% (95% CI: 16.9–20.1) at 5 years (Figure 2b,c).

**Figure 3:**
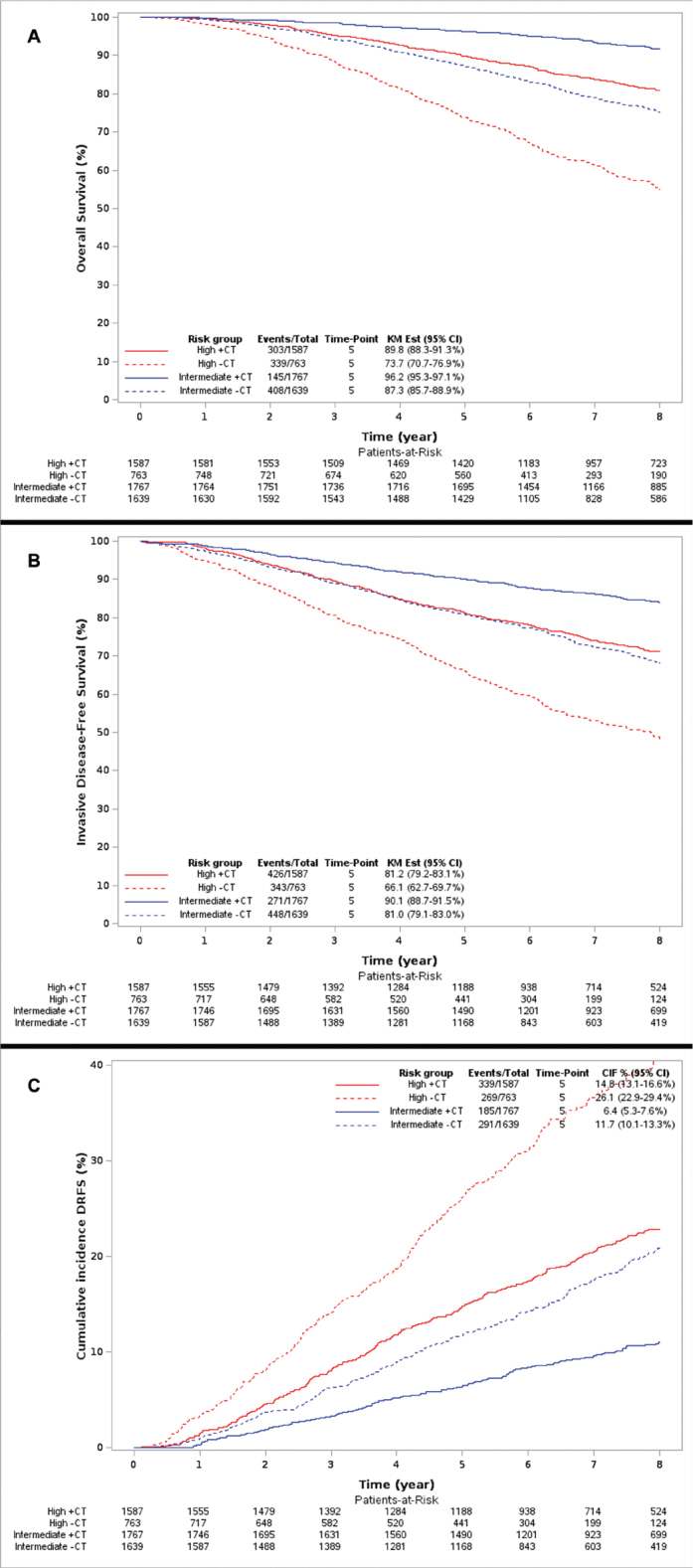
Overall survival (A), invasive disease-free survival (B), and cumulative incidence of distant recurrence-free survival events (C) in the intermediate- and high-risk groups +/- chemotherapy (CT).

Further subgroup analysis of OS, iDFS, and cumulative DRFS in the intermediate- and high-risk groups for those receiving chemotherapy in addition to ET (+CT) and for those only receiving ET (-CT) shows 5-year OS rates of 87.3% (95% CI: 85.7–88.9) in the intermediate-risk group -CT, 73.7% (95% CI: 70.7–76.9) in the high-risk group -CT, 96.2% (95% CI: 95.3–97.1) in the intermediate-risk group +CT, and 89.8% (95% CI: 88.3–91.3) in the high-risk group +CT (Figure 3a).

The 5-year iDFS and cumulative DRFS events rates were 81.0% (95% CI: 79.1–83.0) and 11.7% (95% CI: 10.1–13.3) in the intermediate-risk group -CT, 66.1% (95% CI: 62.7–69.7) and 26.1% (95% CI: 22.9–29.4) in the high-risk group -CT, 90.1% (95% CI: 88.7–91.5) and 6.4% (5.3–7.6) in the intermediate-risk group +CT, and 81.2% (95% CI: 79.2–83.1) and 14.8% (95% CI: 13.1–16.6) in the high-risk group +CT (Figure 3b, c).

**Figure 4:**
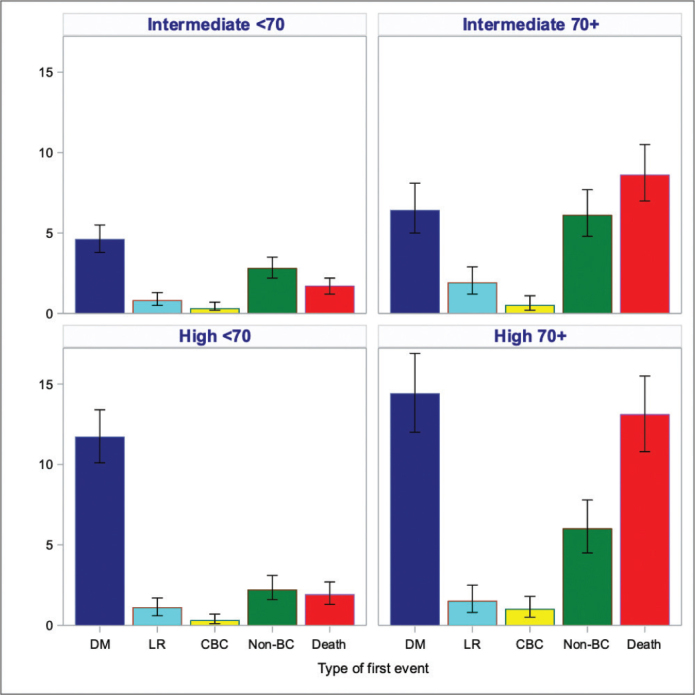
Cumulative incidence (%) at 5 years for specific events in the intermediate- and high-risk groups according to age.

In patients aged < 70 years, distant recurrence was the most frequent first event in both the intermediate- and high-risk groups (Figure 4). In the high-risk group, distant recurrence was also the most frequent first event in patients aged ≥ 70 years, whereas death was the most frequent first event in patients aged ≥70 years in the intermediate-risk group.

Of the 5,788 patients, 77% (95% CI: 75.8–78.2) completed the recommended 5 years of ET (Supplementary Figure 1).

## Discussion

Our study found that patients with ER-positive breast cancer, despite receiving adjuvant ET, demonstrated disappointing OS and iDFS in both intermediate- and high-risk subgroups. For patients under the age of 70, distant recurrence was the most common initial event in both risk groups. This trend continued in the high-risk patients aged 70 and above, with distant recurrence being the most frequent event as well.

Patients who received both chemotherapy and ET had better iDFS, DRFS, and OS compared to those who received endocrine treatment alone. It should be noted, however, that those who received chemotherapy were markedly younger, and tumor characteristics differed between the groups that received and did not receive chemotherapy.

The patients in this study differed in several aspects from the MonarchE and NATALEE clinical trial patients. Specifically, they had a higher median age (62 vs. 51–52 years), a greater proportion of postmenopausal patients (73% vs. 56%), a lower nodal burden (19% vs. 43–60% with N2, N3), and less received chemotherapy (58% vs. 85%).

In the MonarchE trial, nearly one in four patients in the endocrine-alone arm recurred within 5 years. In our study, 23.8% of high-risk patients experienced an iDFS event at 5 years, consistent with data from the GEICAM cohort [6], where 24.8% experienced an iDFS event at 5 years and 43% at 10 years. An Australian real-world study [7] showed that the 5-year and 10-year DFS were 77% and 66% among adjuvant abemaciclib-eligible or high-risk patients and 86% and 77% among the intermediate-risk patients. The intermediate-risk patients in our study had a similar iDFS at 5 years of 85.7%. An American registry study [8] presented 5-year mortality rates of 16.5% for high-risk patients and 7.0% for intermediate-risk patients, similar to our study that showed 5-year mortality rates of 15.5% and 8.1% for high- and intermediate-risk patients, respectively. Our follow-up for OS data ends at 8 years, but real-world data from the Netherlands [9] showed a 10-year OS rate of 63.4% in high-risk patients and 75.1% in intermediate-risk patients. Similarly, a recent Swedish nationwide population-based study presented 10-year OS rates of 65.7% and 64.3% for high-risk patients (MonarchE-eligible and concordant eligible) and 75.5% in intermediate-risk patients (NATALEE-only eligible) [10].

Five-year data are crucial for evaluating the efficacy of adjuvant therapy in ER-positive, HER2-negative EBC [11, 12]. Meta-analyses reveal recurrence rates peak within the first 1–3 years of initiating ET, with one in six patients experiencing recurrence within 5 years [6, 13]. However, long-term follow-up remains essential, as women with ER-positive breast cancer and four to nine positive nodes, for instance, continue to face a significant risk of late recurrence after 5 years of adjuvant ET: Approximately 2.7% per year, or about 13% over the 5–10-year period post-diagnosis [13].

Distant recurrence risk is closely linked to the original tumor-node (TN) status [14]. The recent meta-analysis, which included only patients from randomized trials, showed a significant decrease in the 10-year risk of distant recurrence for women diagnosed after 2000. This improvement is partially attributed to several factors, such as the inclusion of lower-risk patients in trials due to screening, better staging, biomarkers, and advances in systemic adjuvant therapies [15–17].

Despite these improvements, high-risk patients, such as those with T2N2 tumors, still face a recurrence risk of approximately 30% at 10 years [14], which aligns with the high cumulative incidence of DRFS events rate seen in the high-risk group in our study.

While most patients in the MonarchE and NATALEE trials received adjuvant chemotherapy, our real-world data reveal that only half of intermediate-risk and two-thirds of high-risk Danish patients with breast cancer received CT, highlighting a difference in treatment practices for these populations. More than half of the patients in the high-risk group were *allocated* by the national guidelines to CT but were opted out by the treating physician and patient due to unrecorded reasons, but most likely poor performance, high age, and comorbidities. The elevated breast cancer–specific mortality, together with factors such as advanced age, contributes to the increased overall mortality observed in this patient group, thereby explaining the marked difference. This disparity reflects real-world treatment practice [18]. Furthermore, in Denmark, treatment allocation to ET ± CT for postmenopausal patients with up to three positive lymph nodes is guided by the PSI score, which incorporates age as a prognostic determinant [19]. The PSI-score has recently been evaluated in almost 9,000 postmenopausal patients diagnosed with breast cancer between Aug 1, 2013, and Dec 31, 2018 [5]. This analysis showed that among patients in the low-risk group (PSI = 1) 24% had N1 disease. Despite this, those who received adequate ET did not experience excess mortality without adjuvant CT. This suggests that not all patients with N1 disease need the addition of a CDK4/6 inhibitor or a more intensive chemotherapy, provided they adhere to the planned ET. Patients in the high-risk group (PS3-4) had excess mortality despite adjuvant CT in addition to ET.

Nevertheless, the considerable proportion of patients not receiving the allocated chemotherapy regimen underscores the need for more effective and less toxic adjuvant treatment alternatives, including CDK4/6 inhibitors. Evidence from randomized clinical trials assessing the potential of CDK4/6 inhibitors to replace chemotherapy in selected cases is, however, still lacking [20]. The combination of a CDK4/6 inhibitor with ET has been used safely in metastatic settings and has shown comparable efficacy across subgroups of older patients, including patients with multiple or severe comorbidities [21, 22].

Strengths of the study include a large nationwide cohort with a systematic registration of patient characteristics, treatment and adherence to the treatment, and a follow-up of more than 7 years.

In this study, only 77% of patients completed the recommended 5 years of ET, which is similar to other real-world studies with a significant negative effect on survival and DFS for patients with nonadherence [5, 18, 23, 24]. We did not investigate the causes for patients not adhering to ET, but adverse effects have previously been reported by the DBCG as the main cause for discontinuation of ET [18]. Sociodemographic factors are also known causes, such as longer distance to the oncology clinic and unemployment, as well as being of older age (>75 years) or younger (< 40 years) [18, 25]. Inadequate knowledge among healthcare professionals regarding prognosis in intermediate- and high-risk patients, differences in information provided, poor logistics in follow-up programs, and managing severe side effects that compromise quality of life may be factors at cause [25–27].

Introducing CDK4/6 inhibitors into routine clinical practice must include measures to optimize adherence, ensuring that the endocrine backbone is not discontinued due to side effects potentially caused by the CDK4/6 inhibitor. Side effects of CDK4/6 inhibitors include gastrointestinal symptoms and myelosuppression but are generally well tolerated clinically with overall maintained health-related quality of life [28, 29]. In MonarchE, 6.5% of patients in the abemaciclib arm and 1.1% in the ET-alone arm discontinued therapy within 2 years due to side effects [2]. In NATALEE, 19% of patients in the ribociclib arm discontinued treatment with ribociclib due to adverse events at a median follow-up of 34 months compared to 3.3% that discontinued both treatments [3, 30].

In the NATALEE trial, 62.8% completed the 3-year duration of ribociclib compared to 72.2% who completed the 2-year treatment period in MonarchE [4, 31]. Despite high discontinuation rates, iDFS was improved in both studies, indicating that even though compliance generally poses a problem, the outcome still overall improves with treatment.

Approximately one-third of all new patients diagnosed with EBC in Denmark have ER-positive, HER2-negative disease with intermediate- or high-risk features, comparable to other studies [10, 32]. Although many patients may be eligible for a CDK4/6 inhibitor, recurrence will inevitably occur in some cases despite therapy intensification. The potential improvement in outcomes must be weighed against treatment-related toxicity and the number needed to treat, emphasizing the importance of careful patient selection. Ongoing randomized trials aim to elucidate predictive markers to better identify patients most likely to benefit [32].

## Conclusion

Our real-world study shows that a substantial subset of Danish patients with ER-positive, HER2-negative EBC exhibit intermediate- or high-risk disease with poor prognosis, particularly among those not receiving chemotherapy who tended to be older.

Younger patients may also benefit from the addition of CDK4/6 inhibitor as distant recurrence remains the most common initial event for those under the age of 70 years of age, irrespective of their risk group. The addition of a CDK4/6 inhibitor thus appears advantageous to high-risk patients and to intermediate-risk patients where chemotherapy is not tolerated or where chemotherapy in addition to ET is not sufficient, although individual factors must be considered.

## Supplementary Material



## Data Availability

Clinical data included in this study are not publicly available but can be requested for research purposes by contact to the Danish Breast Cancer Group (dbcg.rigshospitalet@regionh.dk).
