# Supplementary material for: Outcomes for patients with high-risk ER-positive, HER2-negative early-stage breast cancer: a Danish real-world study
Source: Acta Oncol. 2025 Nov 12;64:44003. doi: 10.2340/1651-226X.2025.44003 (PMC12625151; doi:10.2340/1651-226X.2025.44003)
Supplement: Supplementary file 1 [file AO-64-44003-s1.pdf]

Supplementary material has been published as submitted. It has not been copyedited, or typeset by Acta Oncologica

T	N	Grade	Stage	Natalee	MonarchE	Current study
T0	N1mic		1b	Excluded	Excluded	Excluded
	N1mac		2a	Included	Excluded	Intermediate
T1	N0		1a	Excluded	Excluded	Excluded
	N1mic	I-II/?	1b	Excluded	Excluded	Excluded
		III		Excluded	Included	High
	N1mac	I-II/?	2a	Included	Excluded	Intermediate
		III		Included	Included	High
T2	N0	I-II/?	2a	Excluded	Excluded	Excluded
		III		Included	Excluded	Intermediate
	N1	I-II/?	2b	Included	Excluded	Intermediate
		III		Included	Included	High
T3	N0		2b	Included	Excluded	Intermediate
	N1		3a	Included	Included	High
Any	N2		3a	Included	Included	High
	N3		3c	Included	Included	High
T4	N0		3b	Included	Excluded	High
	N1-2		3b	Included	Included	High

Included Excluded Intermediate High

Supplementary table 1. Grouping in the current study compared to the NATALEE and MonarchE study. The question mark (?) refers to non-ductal or non-lobar cancers or unknown malignancy grade.
NATALEE trial: Stage II and III disease included.
MonarchE trial: High-risk defined as 4 or more pathologically positive axillary lymph nodes, or 1-3 positive axillary lymph nodes plus either grade 3 tumor, tumor size of 5 cm or greater, or Ki-67 of at least 20%.

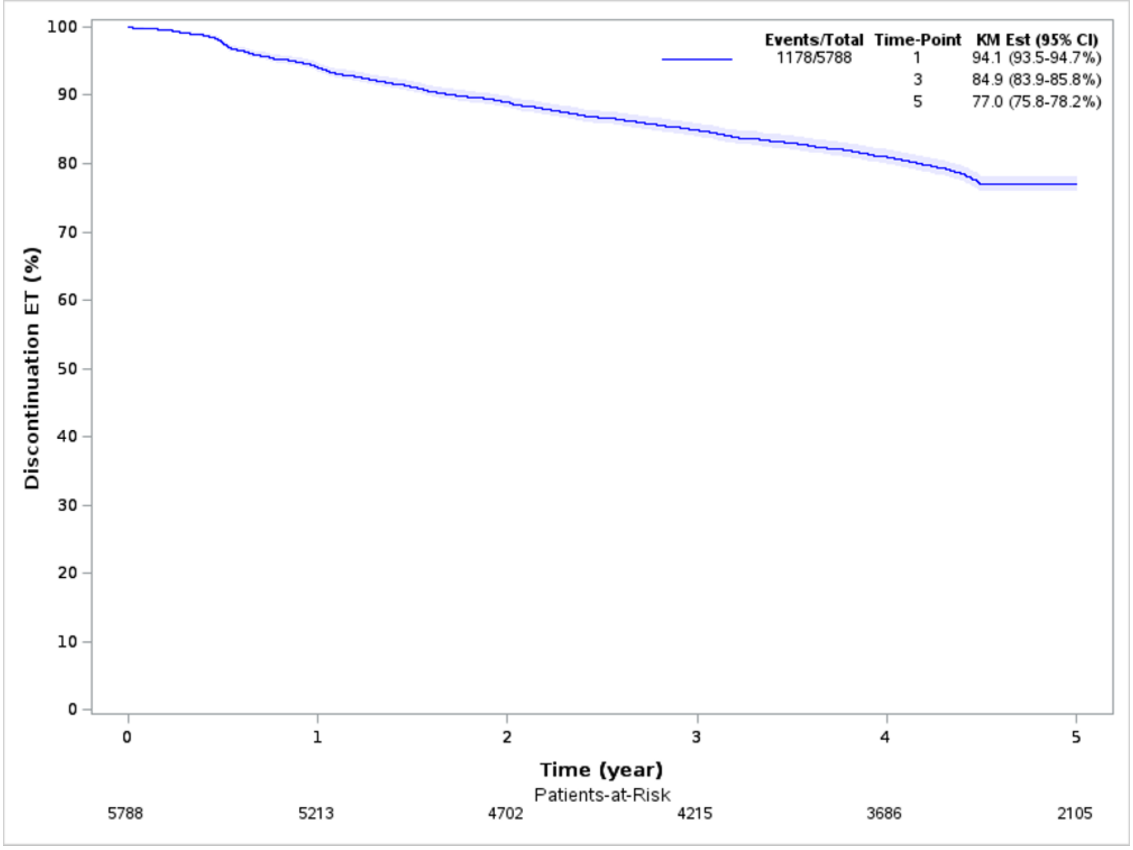

Supplementary figure 1. Adherence to endocrine therapy.
